# Poor Cognitive Function Is Associated with Obstructive Lung Diseases in Taiwanese Adults

**DOI:** 10.3390/ijerph18052344

**Published:** 2021-02-27

**Authors:** Sun-Wung Hsieh, Da-Wei Wu, Chih-Wen Wang, Szu-Chia Chen, Chih-Hsing Hung, Chao-Hung Kuo

**Affiliations:** 1 Department of Neurology, Kaohsiung Medical University Hospital, Kaohsiung Medical University, Kaohsiung 807, Taiwan; circle.6@yahoo.com.tw; 2 Department of Neurology, Kaohsiung Municipal Siaogang Hospital, Kaohsiung Medical University, Kaohsiung 812, Taiwan; 3 Neuroscience Research Center, Kaohsiung Medical University, Kaohsiung 807, Taiwan; 4 Department of Internal Medicine, Kaohsiung Municipal Siaogang Hospital, Kaohsiung Medical University, Kaohsiung 812, Taiwan; chinwin.wang@gmail.com (C.-W.W.); kjh88kmu@gmail.com (C.-H.K.); 5 Division of Pulmonary and Critical Care Medicine, Department of Internal Medicine, Kaohsiung Medical University Hospital, Kaohsiung Medical University, Kaohsiung 807, Taiwan; 6 Doctoral Degree Program of Department of Public Health, College of Health Sciences, Kaohsiung Medical University, Kaohsiung 807, Taiwan; 7 Research Center for Environmental Medicine, Kaohsiung Medical University, Kaohsiung 807, Taiwan; pedhung@gmail.com; 8 Division of Hepatobiliary, Department of Internal Medicine, Kaohsiung Medical University Hospital, Kaohsiung Medical University, Kaohsiung 807, Taiwan; 9 Division of Nephrology, Department of Internal Medicine, Kaohsiung Medical University Hospital, Kaohsiung Medical University, Kaohsiung 807, Taiwan; 10 Faculty of Medicine, College of Medicine, Kaohsiung Medical University, Kaohsiung 807, Taiwan; 11 Department of Pediatrics, Kaohsiung Medical University Hospital, Kaohsiung Medical University, Kaohsiung 807, Taiwan; 12 Department of Pediatrics, Kaohsiung Municipal Siaogang Hospital, Kaohsiung Medical University, Kaohsiung 812, Taiwan; 13 Division of Gastroenterology, Department of Internal Medicine, Kaohsiung Medical University Hospital, Kaohsiung Medical University, Kaohsiung 807, Taiwan

**Keywords:** obstructive and restrictive lung function, cognitive decline, Mini-Mental State Examination

## Abstract

Previous studies have reported an association between the impairment of cognitive performance and lung diseases. However, whether obstructive or restrictive lung diseases have an impact on cognitive function is still inconclusive. We aimed to investigate the association between cognitive function and obstructive or restrictive lung diseases in Taiwanese adults using the Mini-Mental State Examination (MMSE). In this study, we used data from the Taiwan Biobank. Cognitive function was evaluated using the MMSE. Spirometry measurements of forced expiratory volume in 1 s (FEV1) and forced vital capacity (FVC) were obtained to assess lung function. Participants were classified into three groups according to lung function, namely, normal, restrictive, and obstructive lung function. In total, 683 patients enrolled, of whom 357 participants had normal lung function (52.3%), 95 had restrictive lung function (13.9%), and 231 had obstructive lung function (33.8%). Compared to the normal lung function group, the obstructive lung function group was associated with a higher percentage of cognitive impairment (MMSE < 24). In multivariable analysis, a low MMSE score was significantly associated with low FVC, low FEV1, and low FEV1/FVC. Furthermore, a low MMSE score was significantly associated with low FEV1 in the participants with FEV1/FVC < 70%, whereas MMSE was not significantly associated with FVC in the participants with FEV1/FVC ≥ 70%. Our results showed that a low MMSE score was associated with low FEV1, low FVC and low FEV1/FVC. Furthermore, a low MMSE score was associated with obstructive lung diseases but not with restrictive lung diseases.

## 1. Introduction

Mild cognitive impairment (MCI) is a transitional stage between normal aging and dementia, especially Alzheimer’s disease [1]. It is a neurocognitive disease characterized by cognitive impairment exceeding that expected for age and education level, but not significant enough to interfere with instrumental activities of daily living [2]. The prevalence of MCI differs by age as follows: 6.7% for those aged 60–64 years, 8.4% for those aged 65–69 years, 10.1% for those aged 70–74 years, 14.8% for those aged 75–79 years, and 25.2% for those aged 80–84 years [3]. MCI is associated with a 5 to 10% annual conversion rate to dementia [4,5]. In addition, Petersen et al. reported that the cumulative incidence rate of dementia for people with MCI aged over 65 years was as high as 14.9% after two years of follow-up [3]. The exact cause of MCI is unclear, but some measures to help prevent cognitive decline have been proposed, including aerobic exercise, mental activity, and controlling cardiovascular risk factors in patients with MCI [6]. In addition, current research has focused on improving the early detection and treatment of MCI.

Lung diseases are considered to be a determinant of cognitive decline and dementia [7,8]. Pathan et al. concluded that impaired lung function was independently associated with worse cognitive function at baseline and a higher subsequent risk of dementia hospitalization. However, they found no association between lung function and cognitive decline over time [9]. Other studies have reported different and inconsistent results. For example, Weuve et al. provided limited evidence of an inverse association between forced expiratory volume in 1 s (FEV1) and cognitive aging [10]. Moreover, several studies have reported that mid-life lung function can predict psychomotor ability, memory, processing speed, and executive function in mid-life, but only a significant decline in psychomotor ability over time [11,12]. Taken together, these findings point to a decline in cognition and FEV1 with age. Other studies have reported an independent association between FEV1 and cognitive function in all age groups, but the correlations were weak [13,14].

Lung diseases and impaired lung function are preventable, and therefore it is important to identify the modifiable risk factors for MCI. However, whether obstructive or restrictive lung diseases have an impact on cognitive function is unknown. Currently, air pollution is a world-wide public health issue that has reported impacts on respiratory, cardiovascular, and neurological systems [15]. Air pollution has been shown to pose threats to health, and it has been linked to many diseases, including cardiovascular diseases, chronic obstructive pulmonary diseases (COPD), and even autoimmune diseases [16]. Emerging evidence has also shown associations between air pollution and cognitive decline and neurological disorders, such as Alzheimer’s disease and Parkinson’s disease [17], which will increase the burden on our aging society. Therefore, the aim of this study was to investigate associations between cognitive function and different types of lung diseases with confounding factors such as lifestyle and cardiovascular risks. We investigated cognitive function using the Mini-Mental State Examination (MMSE) in individuals with lung diseases using data from the Taiwan Biobank (TWB) to clarify the clinical interpretation.

## 2. Methods

### 2.1. The TWB

The TWB was created with the aim of recording lifestyle and genomic data of residents in Taiwan, where it is currently the largest biobank supported by the Taiwanese government [18,19]. The TWB includes the information of community-based volunteers with no prior history of cancer and who are aged between 30 and 70 years. All of these volunteers provide written informed consent, blood samples, and complete questionnaires during face-to-face interviews with researchers from the TWB. In addition, all of the participants in the TWB undergo physical examinations. In this study, we included 5000 participants who were registered in the TWB as of April 2014.

The data stored in the TWB include body height, body weight, and personal and lifestyle factors. In this study, we defined regular exercise as participating for at least 30 min three times a week in activities including jogging, hiking, playing a sport, yoga, swimming, cycling, and computer/console-based exercise games. However, work-related activities such as physical or manual work were not classified as being “exercise” in this study.

### 2.2. Demographic, Laboratory, and Medical Data

Demographic characteristics (sex and age), smoking habits, medical history (hypertension, asthma, emphysema or bronchitis, and diabetes mellitus (DM)), lifestyle factors (regular exercise and midnight snacking habits), systolic blood pressure (SBP), diastolic blood pressure (DBP), education duration, and laboratory data (estimated glomerular filtration rate (eGFR), triglycerides, hemoglobin, fasting glucose, total cholesterol, and uric acid) were recorded at baseline. EGFR was calculated using the modification of diet in renal disease 4-variable equation [20]. Body mass index (BMI) was calculated as weight (kg)/height (m)^2^.

### 2.3. Evaluation of Cognitive Function

We assessed the cognitive function of the subjects using the MMSE [21]. The MMSE is used as a screening tool to assess cognitive impairment, with a low score indicating the need for further evaluations. The total MMSE score was calculated by summing up subscale scores, with a maximum score of 30 points. One hundred and fifty-four participants with complete MMSE measurements during the enrollment period were included in this study.

### 2.4. Spirometry Measurements

Spirometry measurements (in L) of FEV1 and forced vital capacity (FVC) were obtained. Spirometry tests were conducted using a MicroLab spirometer and Spida 5 software (Micro Medical Ltd., Rochester, Kent, UK) by a trained technician according to the 2005 technical standards of the American Thoracic Society and the European Respiratory Society [22]. We performed three lung function tests in each participant, all of which met the quality criteria standards (i.e., with differences within 5% or 100 mL), and the best result of the three tests was used for analysis. FVC-predicted (or FVC%-predicted) and FEV1-predicted (or FEV1%-predicted) values were calculated by dividing the measurements by reference values, which were calculated according to formulas derived from the general population based on Asian ethnicity, sex, age, and height. The formulas for this population were entered into spirometry software, and the details of individual participants were also entered to yield percent-predicted values. There were no post-bronchodilator measurements. A total of 1054 participants were screened for this study, of whom 371 did not have complete spirometry measurements during the enrollment period and were excluded, while the remaining 683 participants with complete spirometry measurements were included.

### 2.5. Ethics Statement

The Institutional Review Board on Biomedical Science Research/IRB-BM, Academia Sinica, Taiwan, and the Ethics and Governance Council of the TWB, Taiwan, provided ethical approval for the TWB. Each participant provided written informed consent in accordance with institutional requirements, and this study was conducted in accordance with the principles of the Declaration of Helsinki. In addition, the Institutional Review Board of Kaohsiung Medical University Hospital approved this study (KMUHIRB-E(I)-20180242).

### 2.6. Statistical Analysis

Data are presented as percentages, means ± standard deviations, or median (25^th^–75^th^ percentile) for triglycerides. An MMSE cut-off score of 24 was used to classify the severity of cognitive impairment. The study participants were classified into three groups according to lung function. One-way analysis of variance was used to compare differences among groups, followed by a Bonferroni-adjusted post hoc test. Multivariate stepwise linear regression analysis was used to identify factors associated with FVC, FEV1, and FEV1/FVC. A *p* value of less than 0.05 was considered to indicate a statistically significant difference. Statistical analysis was performed using SPSS version 19.0 for Windows (SPSS Inc., Chicago, IL, USA).

## 3. Results

The mean age of the 683 enrolled participants was 63.9 ± 2.8 years, and included 337 males and 346 females. The participants were stratified into three groups according to lung function as follows: normal lung function (FEV1/FVC ≥ 70% and FVC predicted ≥ 80%; *n* = 357, 52.3%), restrictive lung function (FEV1/FVC ≥ 70% and FVC-predicted < 80%; *n* = 95, 13.9%), and obstructive lung function (FEV1/FVC < 70%; *n* = 231; 33.8%). A comparison of the clinical characteristics among these three groups is shown in Table 1. Compared to the participants with normal lung function, those with restrictive lung function were older, had higher SBP, lower FVC, lower FVC-predicted, lower FEV1, and lower FEV1-predicted. On the other hand, compared to the participants with normal lung function, those with obstructive lung function were more predominantly female, and more had an MMSE total score <24, had lower FVC, lower FVC-predicted, lower FEV1, lower FEV1-predicted, and lower FEV1/FVC.

### 3.1. Correlations between MMSE with FVC, FEV1, and FEV1/FVC in All Participants

Table 2 shows the determinants of FVC, FEV1, and FEV1/FVC in all participants according to multivariable stepwise linear regression analysis after adjusting for sex, age, hypertension, smoking history, DM, a history of asthma, emphysema or bronchitis, lifestyle factors of regular exercise and midnight snacking habits, BMI, total cholesterol, log triglycerides, fasting glucose, hemoglobin, eGFR, uric acid, SBP, DBP and MMSE score. The results showed that an older age, female sex, history of smoking, asthma, emphysema or bronchitis, not participating in regular exercise, high SBP, high eGFR and low MMSE score (unstandardized coefficient β = 0.018; *p* = 0.008) were significantly associated with low FVC. In addition, an older age, female sex, smoking history, high SBP and low MMSE score (unstandardized coefficient β = 0.021; *p* = 0.007) were significantly associated with low FEV1. Finally, female sex, high total cholesterol, and low MMSE score (unstandardized coefficient β = 0.475; *p* = 0.049) were significantly associated with low FEV1/FVC. For the sample size *n* = 683, the study is almost balanced with respect to Type I and Type II error rates, with α = 0.05 and β = 1 − 0.999 = 0.0001 (power test = 100%).

We further performed multivariable analysis after adjustment of education level (o; ≦6 and >6 years), and found low MMSE score was still significantly associated with low FVC (unstandardized coefficient β = 0.017; *p* = 0.009) and low FEV1 (unstandardized coefficient β = 0.021; *p* = 0.008).

### 3.2. Correlation between MMSE and FEV1 in the Participants with FEV1/FVC < 70%

Table 3 shows the determinants of FEV1 in the study participants with FEV1/FVC < 70% using multivariable stepwise linear regression analysis. The results showed that female sex and low MMSE score (unstandardized coefficient β = 0.019; *p* = 0.041) were significantly associated with low FEV1. For the sample size *n* = 231, the study is almost balanced with respect to Type I and Type II error rates, with α = 0.05 and β = 1 − 0.991 = 0 (power test = 99.1%).

We further performed multivariable analysis after adjustment of education level, and found low MMSE score was still significantly associated with low FEV1 (unstandardized coefficient β = 0.019; *p* = 0.041).

### 3.3. Correlation between MMSE and FVC in the Participants with FEV1/FVC ≥ 70%

Table 4 shows the determinants of FVC in the study participants with FEV1/FVC ≥ 70% using multivariable stepwise linear regression analysis. The results showed that older age, female sex, not participating in regular exercise, and high SBP were significantly associated with low FVC, whereas MMSE score was not significantly associated with low FVC. For the sample size *n* = 452, the study is almost balanced with respect to Type I and Type II error rates, with α = 0.05 and β = 1 − 0.999 = 0.001 (power test = 100%).

## 4. Discussion

In this study, we found that the presence of obstructive, but not restrictive, lung diseases was associated with a higher percentage of cognitive impairment (MMSE < 24). Overall, a low MMSE score was independently associated with worse lung function as indicated by low FVC, low FEV1, and low FEV1/FVC. Furthermore, a low MMSE score was significantly associated with low FEV1 in the participants with obstructive lung function, whereas MMSE was not significantly associated with FVC in the participants with restrictive lung function.

Increasing evidence has shown an association between compromised lung health with dementia and a decline in cognitive ability. Lutsey et al. concluded that lung diseases, and mainly restrictive but also to a lesser degree obstructive diseases, were associated with an increased risk of incident dementia and MCI in a 27-year community-based cohort study. Such findings have also been reported in patients with Alzheimer’s disease, vascular dementia, and even nonsmokers [23]. The findings of our study are different from previous studies in that we found that obstructive, but not restrictive, lung diseases were associated with cognitive impairment. For people with restrictive lung diseases, older age, female sex, hypertension, and not participating in regular exercise were associated with worse lung function as indicated by FVC. We focused mainly on the influence of cognitive function rather than incident dementia. In comparison to Lutsey’s study, [23] in which the average age of the participants was 54.2  ±  5.8 years, our study included older participants with a mean age of 63.9 ± 2.8 years. In addition, our study participants were East Asian, whereas those in Lutsey’s study were mostly Caucasian and 25.9% were African American. Moreover, the neuropsychological assessments used to assess dementia and MCI in Lutsey’s study were adopted from the Atherosclerosis Risk in Communities Neurocognitive Study, [24] and involved detailed neurocognitive assessments, neurologic examinations, brain imaging, validated telephone-based cognitive assessments, and modified telephone interviews to evaluate cognitive status or hospitalization codes. In comparison, we used the MMSE as the only cognitive assessment tool which was administered by well-trained staff. These differences may partially explain the difference in results between the two studies.

The important finding of the present study was that a low MMSE score was associated with low FVC, low FEV1, and low FEV1/FVC. Furthermore, a low MMSE score was associated with obstructive lung function, but not restrictive lung function. Obstructive and restrictive lung diseases have different pathophysiologies. Obstructive lung diseases include asthma, bronchiectasis, bronchitis, and COPD. Previous studies have reported that COPD was associated with a nearly 80% higher risk of developing MCI over five years [25], and MCI or dementia over 25 years [26]. The duration of COPD has also been associated with the risk of MCI, [25] and a clinical history of COPD has also been associated with a decline in cognitive performance over time [27]. However, Pathan et al. did not find an association between the presence of obstructive lung diseases and a greater risk of dementia hospitalization [9]. COPD and asthma patients generally have higher rate of comorbidities, including cardiovascular-, bone-, and other smoking-related conditions at baseline [28]. These comorbidities in COPD patients are independent of smoking and traditional risk factors, and more cardiovascular events have been shown to contribute to a worse cognitive decline in COPD patients [29,30,31]. Patients with COPD are at higher risk for atherosclerotic disease [32]. As in our study, the cholesterol levels were higher in the obstructive group than in the normal group (205.4 ± 36.8 vs. 199.6 ± 36.4), which indicated a higher risk for cerebrovascular disease in the obstructive group. Such findings explained the higher risks of impairment of cognitive performance in patients with obstructive lung disease. It is now well established that atherosclerotic disease contributes significantly to both morbidity and mortality in COPD. Shared risk factors for atherosclerotic disease and COPD, such as smoking, low socioeconomic class, and a sedentary lifestyle contribute to the natural history of each of these conditions. Restrictive lung diseases are characterized by limited lung expansion, resulting in reduced lung volume, ventilation–perfusion mismatch, and hypoxemia. Yaffe et al. demonstrated that hypoxemia during sleep increased the risk of MCI or dementia over a 4.7-year follow-up period [33]. Chronic constant hypoxemia due to restrictive or obstructive lung diseases has been reported to affect neurological function through systemic inflammation, oxidative stress, physiologic stress, sympathetic nervous system activation, cerebral arterial stiffness, and small-vessel damage [7,34].

The impact of lung diseases on the risk of dementia and MCI may differ by race. COPD has been associated with the risk of dementia and MCI in black people, whereas restrictive impairment has been associated with cognitive impairment in white people [23]. Differences in the underlying specific pathologies between obstructive and restrictive impairment patterns have also been reported to vary by race [35,36]. This may explain the impact of different lung diseases on the risk of MCI between Caucasians and the East Asian population observed in our study.

There are important strengths to our study, including the large community-based sample of healthy individuals with no history of cancer, objective ascertainment of lung function using standard protocols, comprehensive neurocognitive assessment using the MMSE, and controlling for confounding factors including lifestyle, smoking, and cardiovascular risks. However, there are also several limitations. First, this was a cross-sectional study. We did not perform the evaluation for how long patients had the lung disease. Therefore, we could not evaluate the causal relationship between lung disease and cognitive function. Further longitudinal studies are warranted to investigate the risk of incident dementia. Second, relatively few cases had restrictive lung diseases, which may have caused bias in the estimations. Third, hypoxemia could be the missing link in our study, however, we lacked data on oxygen levels. Fourth, the MMSE score in our study was not adjusted for sensory impairment. Finally, the use of medications such as hypnotics was not analyzed in this study, and this may have influenced the risk of MCI.

In conclusion, we found that cognitive decline was associated with worse lung function as indicated by low FVC, low FEV1, and low FEV1/FVC. Furthermore, cognitive decline was mainly associated with obstructive lung diseases, but not restrictive lung diseases in our Taiwanese adult participants.

## Figures and Tables

**Table 1:** Comparison of clinical characteristics among participants according to lung function.

Characteristics	Normal (*n* = 357)	Restrictive (*n* = 95)	Obstructive (*n* = 231)	*p*
Age (year)	63.9 ± 2.8	64.9 ± 2.8 *	64.1 ± 3.0	0.011
Male gender (%)	52.4	56.8	41.6 *^,†^	0.011
Smoking history (%)	23.5	34.7	25.1	0.082
DM (%)	10.1	16.8	12.1	0.186
Hypertension (%)	22.7	30.5	22.5	0.244
Asthma history (%)	3.4	6.3	4.8	0.399
Emphysema or bronchitis history (%)	3.4	2.1	5.2	0.340
Regular exercise habits (%)	73.7	61.1	69.7	0.053
Midnight snack habits (%)	19.9	21.1	16.9	0.573
BMI (kg/m^2^)	24.3 ± 3.0	24.7 ± 3.5	24.1 ± 2.9	0.242
SBP (mmHg)	124.2 ± 16.1	130.2 ± 19.3 *	125.9 ± 17.8	0.010
DBP (mmHg)	72.2 ± 10.7	73.3 ± 11.5	71.5 ± 11.1	0.373
Laboratory parameters				
Fasting glucose (mg/dL)	101.2 ± 21.2	101.3 ± 18.9	103.2 ± 26.0	0.569
Triglyceride (mg/dL)	100 (72-138.5)	105 (80-141)	101 (74-131)	0.468
Total cholesterol (mg/dL)	199.6 ± 36.4	197.2 ± 39.6	205.4 ± 36.8	0.093
Hemoglobin (g/dL)	14.1 ± 1.4	14.1 ± 1.5	13.9 ± 1.3	0.279
eGFR (mL/min/1.73 m^2^)	86.8 ± 27.2	85.5 ± 34.4	90.6 ± 27.2	0.188
Uric acid (mg/dL)	5.7 ± 1.4	6.1 ± 1.5	5.6 ± 1.4 ^†^	0.036
MMSE	27.1 ± 2.5	26.7 ± 2.8	26.5 ± 3.1	0.052
MMSE < 24	7.8	12.6	16.9 *	0.003
Lung function				
FVC (L)	2.8 ± 0.6	2.1 ± 0.5 *	2.4 ± 0.7 *^,†^	<0.001
FVC-predicted (%)	95.0 ± 10.8	71.3 ± 8.5 *	87.3 ± 17.4 *^†^	<0.001
FEV1 (L)	2.3 ± 0.5	1.7 ± 0.4 *	1.3 ± 0.5 *^,†^	<0.001
FEV1-predicted (%)	101.2 ± 13.7	78.2 ± 12.0 *	59.8 ± 19.3 *^,†^	<0.001
FEV1/FVC (%)	82.5 ± 5.8	82.6 ± 6.4	52.8 ± 13.3 *^,†^	<0.001

Abbreviations: DM, diabetes mellitus; BMI, body mass index; SBP, systolic blood pressure; DBP, diastolic blood pressure; eGFR, estimated glomerular filtration rate; MMSE, Mini-Mental State Examination; FVC, forced vital capacity, FEV1, forced expiratory volume in 1 s. Normal lung function was defined as FEV1/FVC ≥ 70% and FVC-predicted ≥ 80%; restrictive lung function was defined as FEV1/FVC ≥ 70% and FVC-predicted < 80%; obstructive lung function was defined as FEV1/FVC < 70%. * *p* < 0.05 compared with normal lung function; ^†^ *p* < 0.05 compared with restrictive lung function.

**Table 2:** Association of MMSE with FVC, FEV1 and FEV1/FVC using multivariable stepwise linear regression analysis in all participants (*n* = 683).

Lung Function	Multivariable Stepwise	
	Unstandardized Coefficient β (95% CI)	*p*
FVC (L)		
Age (per 1 year)	−0.033 (−0.046, −0.021)	<0.001
Male vs. female	0.899 (0.798, 1.001)	<0.001
Smoking history	−0.103 (−0.196, −0.010)	0.030
Asthma history	−0.240 (−0.413, −0.067)	0.007
Emphysema or bronchitis history	−0.213 (−0.396, −0.031)	0.002
Regular exercise habits	0.097 (0.020, 0.174)	0.014
SBP (per 1 mmHg)	−0.002 (−0.004, 0)	0.019
eGFR (per 1 mL/min/1.73 m^2^)	−0.002 (−0.003, 0)	0.036
MMSE (per 1 score)	0.018 (0.005, 0.030)	0.008
FEV1 (L)		
Age (per 1 year)	−0.018 (−0.033, −0.004)	0.013
Male vs. female	0.817 (0.721, 0.914)	<0.001
Smoking history	−0.151 (−0.261, −0.042)	0.007
SBP (per 1 mmHg)	−0.003 (−0.005, 0)	0.031
MMSE (per 1 score)	0.021 (0.006, 0.036)	0.007
FEV1/FVC (%)		
Male vs. female	4.593 (0.451, 8.735)	0.030
Total cholesterol (per 1 mg/dL)	−0.044 (−0.081, −0.006)	0.023
MMSE (per 1 score)	0.475 (0.003, 0.948)	0.049

Values expressed as unstandardized coefficient β and 95% confidence interval (CI). Abbreviations are the same as in Table 1. Multivariable model: adjusted for age, sex, smoking history, DM, hypertension, asthma history, emphysema or bronchitis history, lifestyle with regular exercise and midnight snack habits, BMI, SBP, DBP, fasting glucose, log triglyceride, total cholesterol, hemoglobin, eGFR, uric acid, and MMSE.

**Table 3:** Association of MMSE with FEV1 using multivariable stepwise linear regression analysis in participants with FEV1/FVC < 70% (*n* = 231).

FEV1 (L)	Multivariable Stepwise	
	Unstandardized Coefficient β (95% CI)	*p*
Male vs. female	0.526 (0.414, 0.639)	<0.001
MMSE (per 1 score)	0.019 (0.001, 0.037)	0.041

Values expressed as unstandardized coefficient β and 95% confidence interval (CI). Abbreviations are the same as in Table 1. Multivariable model: adjusted for age, sex, smoking history, DM, hypertension, asthma history, emphysema or bronchitis history, lifestyle with regular exercise and midnight snack habits, BMI, SBP, DBP, fasting glucose, log triglyceride, total cholesterol, hemoglobin, eGFR, uric acid and MMSE.

**Table 4:** Association of MMSE with FVC using multivariable stepwise linear regression analysis in participants with FEV1/FVC ≥ 70% (*n* = 452).

FVC (L)	Multivariable Stepwise	
	Unstandardized Coefficient β (95% CI)	*p*
Age (per 1 year)	−0.032 (−0.047, −0.018)	<0.001
Male vs. female	0.906 (0.824, 0.989)	<0.001
SBP ( per 1 mmHg)	−0.004 (−0.006, −0.001)	0.004
Regular exercise habits	0.123 (0.033, 0.213)	0.008

Values expressed as unstandardized coefficient β and 95% confidence interval (CI). Abbreviations are the same as in Table 1. Multivariable model: adjusted for age, sex, smoking history, DM, hypertension, asthma history, emphysema or bronchitis history, lifestyle with regular exercise and midnight snack habits, BMI, SBP, DBP, fasting glucose, log triglyceride, total cholesterol, hemoglobin, eGFR, uric acid and MMSE.

## Data Availability

The data underlying this study is from the Taiwan Biobank. Due to restrictions placed on the data by the Personal Information Protection Act of Taiwan, the minimal data set cannot be made publicly available. Data may be available upon request to interested researchers. Please send data requests to: Szu-Chia Chen, PhD, MD. Division of Nephrology, Department of Internal Medicine, Kaohsiung Medical University Hospital, Kaohsiung Medical University.
